# Ultrathin Air-Stable n-Type Organic Phototransistor Array for Conformal Optoelectronics

**DOI:** 10.1038/s41598-018-35062-7

**Published:** 2018-11-09

**Authors:** Meiling Liu, Haiting Wang, Qingxin Tang, Xiaoli Zhao, Yanhong Tong, Yichun Liu

**Affiliations:** Key Laboratory of UV Light Emitting Materials and Technology under Ministry of Education, Northeast Normal University, Changchun, 130024 P. R. China

## Abstract

Development of conformal *n*-channel organic phototransistor (OPT) array is urgent for future applications of organic complementary circuits in portable and wearable electronics and optoelectronics. In this work, the ultrathin conformal OPT array based on air-stable *n*-type PTCDI-C_13_H_27_ was fabricated. The OPT array shows excellent electrical and photoelectrical performance, good device uniformity, and remains stable in electron mobility by 83% after 90 days compared to the initial values. Eventhough mobility, on-state current, off-state current, and photocurrent of PTCDI-C_13_H_27_ thin film phototransistor show slight decrease with the decreased bending radius, the device still remains the stable photosensitivity as high as 10^4^ when the device is freely adhered on the 2D surfaces and 3D hemispherical sphere, which is in a class with the highest photosensitivity for perylene diimide derivatives. These results present the promising application potential of our conformable air-stable *n*-type PTCDI-C_13_H_27_ OPTs as the photodetection system of curved artificial compound eyes in wearable and portable electronics and optoelectronics.

## Introduction

Conformal organic optoelectronic devices are attracting a great deal of interest for use in wearable and portable optoelectronics such as flexible conformable displays, artificial compound eyes, artificial retina, and photoplethysmogram (PPG) sensors^1–5^. They show the enormous advantages in flexibility, light weight, good conformability onto rough surfaces or nonplanar objects^5–7^. Among versatile organic optoelectronic devices, organic phototransistor (OPT) is very appealing because of its outstanding advantage in the combination of light detection, light switching and signal magnification in a single device^8–12^. Compared with photodiode, the phototransistor typically has higher photosensitivity and lower noise current owing to the presence of an additional gate electrode for amplified photogenerated electrical signals^13–17^.

Until now, only two research groups have reported the conformal OPTs^3,4^. For example, *Zhao et al*. developed a poly(N-alkyl diketo-pyrrolo-pyrrole dithienylthieno[3, 2-b]thiophene) (DPP-DTT) /[6,6]-phenyl-C61-butyric acid methylester (PCBM) phototransistor with thickness over 2 μm that allowed the device to be transferred directly onto human arm and finger^3^. Recently, *Chu et al*. prepared a near 3-μm-thick conformal 2,7-dioctyl[1]-benzothieno[3,2-b] benzothiophene (C8-BTBT)/polylactide (PLA) phototransistor that can be adhered onto a glove^4^. So far, all reports on conformable OPTs have utilized *p*-channel materials, mainly due to lack of *n*-channel organic materials with air stability and good performance^18–20^. The instability of organic radical anions in the presence of oxygen and water, or the electron traps at interfaces, is extensively believed to affect the stability of *n*-channel organic transistors^21,22^. In fact, the development of conformable OPTs based on *n*-channel organic material is extremely important for the fabrication of complementary electronics and optoelectronic circuits, which provides various advantages such as high operational stability, easily controlled photoswitching voltages, high photosensitivity and responsivity^23–25^. Therefore, it is urgent to develop the conformal *n*-channel OPTs for future applications in portable and wearable organic optoelectronics.

Herein, we present the ultrathin conformable OPT arrays on the polyvinyl alcohol (PVA) supporting layer, in which the air-stable *n*-type PTCDI-C_13_H_27_ thin film serves as the active layer, PMMA serves as dielectric layer, and the thickness of the entire OPT is only~830 nm. From the view of molecular design, PTCDI-C_13_H_27_ is one of perylene derivatives that is favorable for the good stability in *n*-type organic transistors^20,26^. At the same time, PMMA is used as the dielectric of the transistor because its hydrophobic nature has been extensively shown to be favorable for good device stability^27,28^. Based on such a stable *n*-type organic transistor, we show the potential of PTCDI-C_13_H_27_ in fabrication of *n*-type conformal OPT array, and the potential application of the devices for future wearable and portable electronics and optoelectronics.

## Results and Discussion

**Figure 1:**
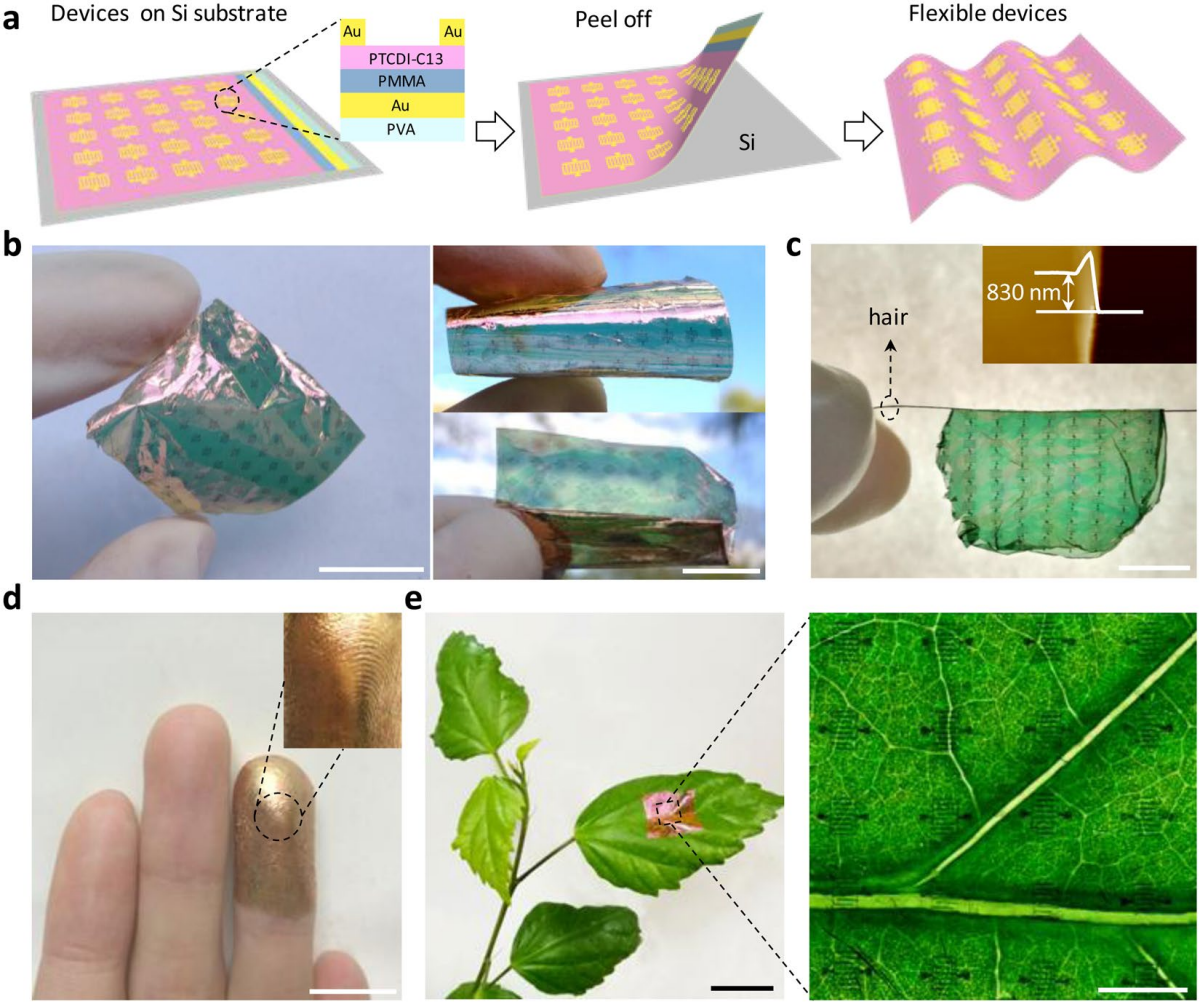
Ultra-flexible and conformable organic phototransistor array based on PTCDI-C_13_H_27_ thin film. (**a**) Schematic images showing the flexible compatible device array peeled from Si substrate. The inset shows the cross-sectional schematic illustration of a single device. (**b**) Photograph of 10 × 11 device array with the area size of 3 × 3 cm^2^ adhered onto a 10-µm commercialized plastic wrap. (**c**) Photograph of device array sustained by a hair. AFM in the inset indicates the thickness of the device array at only ~830 nm. (**d**) Photograph of the device adhered on loops and whorls on human finger. (**e**) Photograph of the device adhered to a plant leave. The right image shows the detailed devices on the plant leave. Scale bar: 1 cm.

Figure 1a presents the schematic illustration of a flexible conformable OPT array with bottom-gate top-contact configuration. The schematic cross-section image in Fig. 1 clearly shows the device structure of OPT. In our experiments, PTCDI-C_13_H_27_ was selected as the semiconductor active material of the *n*-type organic thin film transistor (OTFT), owing to its outstanding air stability and high electron transport capability^29,30^. The flexible device array was peeled from Si substrate as shown in Fig. 1a. The details of fabrication process are given in Experimental Section. Figure 1b shows a 10 × 11 device array with the area size of 3 × 3 cm^2^ that is adhered onto a 10-µm-thickness commercialized plastic wrap. Good flexibility can be observed and the device array is bendable and foldable. The AFM image in the inset of Fig. 1c shows that the thickness of the device is only ~830 nm. Such an ultrathin device array is extremely light, so that it can be sustained by a hair as shown in Fig. 1c. The ultrathin thickness also makes the device array well conform onto different curved objects, for example, human finger (Fig. 1d) and plant leave (Fig. 1e). The veins on the human finger and plant leave can be clearly observed, presenting the promising potential of the ultrathin OPT array for wearable and portable electronics and optoelectronics.

**Figure 2:**
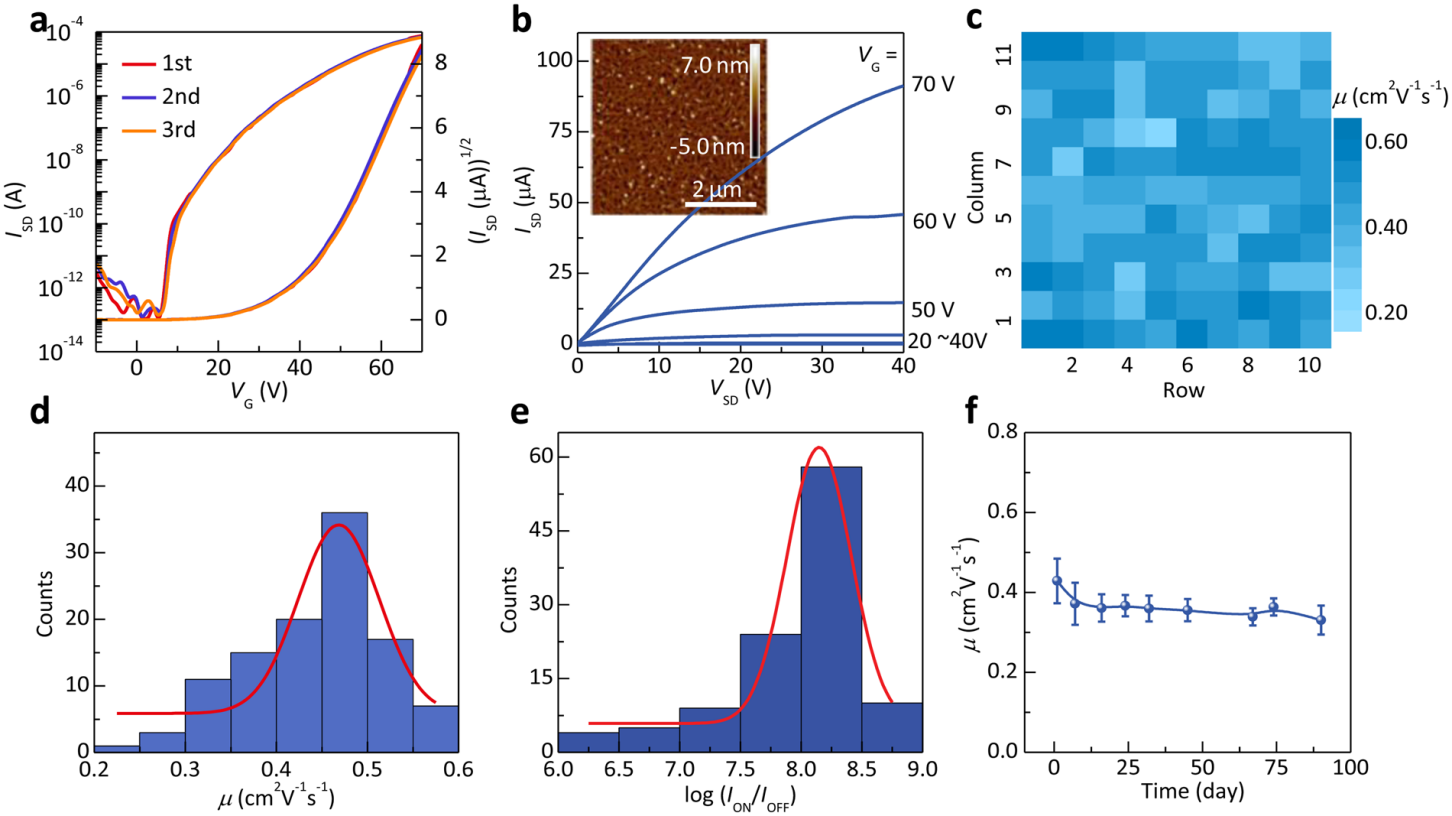
Electrical properties of the PTCDI-C_13_H_27_ thin-film transistor: (**a**,**b**) Typical transfer and output curves of the device. (**a**) Shows the multi-measured results. The inset is AFM image of 5-nm-thick PTCDI-C13 thin films. (**c**) Field-effect mobility mapping of the 10 × 11 phototransistor array. (**d**,**e**) Electron mobility and log(*I*_on_/*I*_off_) distribution of 110 transistors. (**f**) Electron mobility change with time for 90 days in ambient condition from 10 devices.

As organic electronic and optoelectronic devices towards practical applications, the success rate of large-area device fabrication and the performance distribution are the crucial factors. Here, a 10 × 11 PTCDI-C_13_H_27_ thin film phototransistor array was fabricated and its performance distribution was investigated. Figure 2a,b show the typical transfer and output characteristics of the PTCDI-C_13_H_27_ thin film devices, respectively. In a typical *n*-channel operating mode, a positive gate bias induces the accumulation of electron carriers at the interface between active layer and dielectric layer. To characterize operational stability of the ultra-flexible transistors, the multi-measured results are shown in Fig. 2a, which present the excellent stability with overlapped curves. And as shown in the inset of Fig. 2b, the tapping-mode atomic force microscopy (AFM) images of 5-nm PTCDI-C13 thin films deposited onto PMMA dielectric layer show the average grain sizes of ~600 nm. The large grain size of organic semconductor thin film near the channel is favorable for the high charge-carrier mobilities^29^. As a crucial parameter, the field effect mobility was calculated by the following equation: $\mu =\frac{2L}{W{C}_{i}}{(\frac{\partial \sqrt{{I}_{SD}}}{\partial {V}_{GS}})}^{2},$ where *L* and *W* are channel length and width, respectively, and *C*_*i*_ is specific capacitance of the gate dielectric. Color map of the mobility of the OTFT array presents good uniformity, which is expressed by the spatial distribution of the transistor performance (Fig. 2c). The statistical results of Fig. 2d,e show the mobility (*µ*) and current on/off ratio (*I*_on_/*I*_off_) distribution, respectively. Our results show device yield as high as 100%. All transistors show the mobility higher than 0.2 cm^2^ V^−1^ s^−1^. The average mobility is 0.444 cm^2^ V^−1^ s^−1^ with a standard deviation of 0.076 cm^2^ V^−1^ s^−1^. The highest mobility reaches 0.58 cm^2^ V^−1^ s^−1^ in saturation regime. This value is in class with the highest mobility for PTCDI-C_13_H_27_ transistors^29,30^. The other crucial parameter, the current on/off ratio, is used to evaluate the switch and amplification characteristics of transistor. It is a promising result that the *I*_on_/*I*_off_ of our devices is focused on 10^8^. 61.8% of transistors exceed 10^8^, and the maximum *I*_on_/*I*_off_ is as high as 10^9^. For OPTs, the stable dark state is vital for practical applications. Here, the variation and distribution of electron mobility in air ambient are observed without any encapsulation layer. As shown in Fig. 2f, statistically, all 10 measured transistors remain stable in electron mobility by 83% after 90 days compared to the initial values. Therefore, the fabricated devices show the excellent uniformity with 100% success rate and good stability. According to previous reports and our results [Fig. S1], we attribute the good device stability to the molecular design of PTCDI–C_13_H_27_ and hydrophobic nature for the decreased trap density in PMMA dielectric^22,31^. These properties can offer the basic for further study on photoelectric properties.

**Figure 3:**
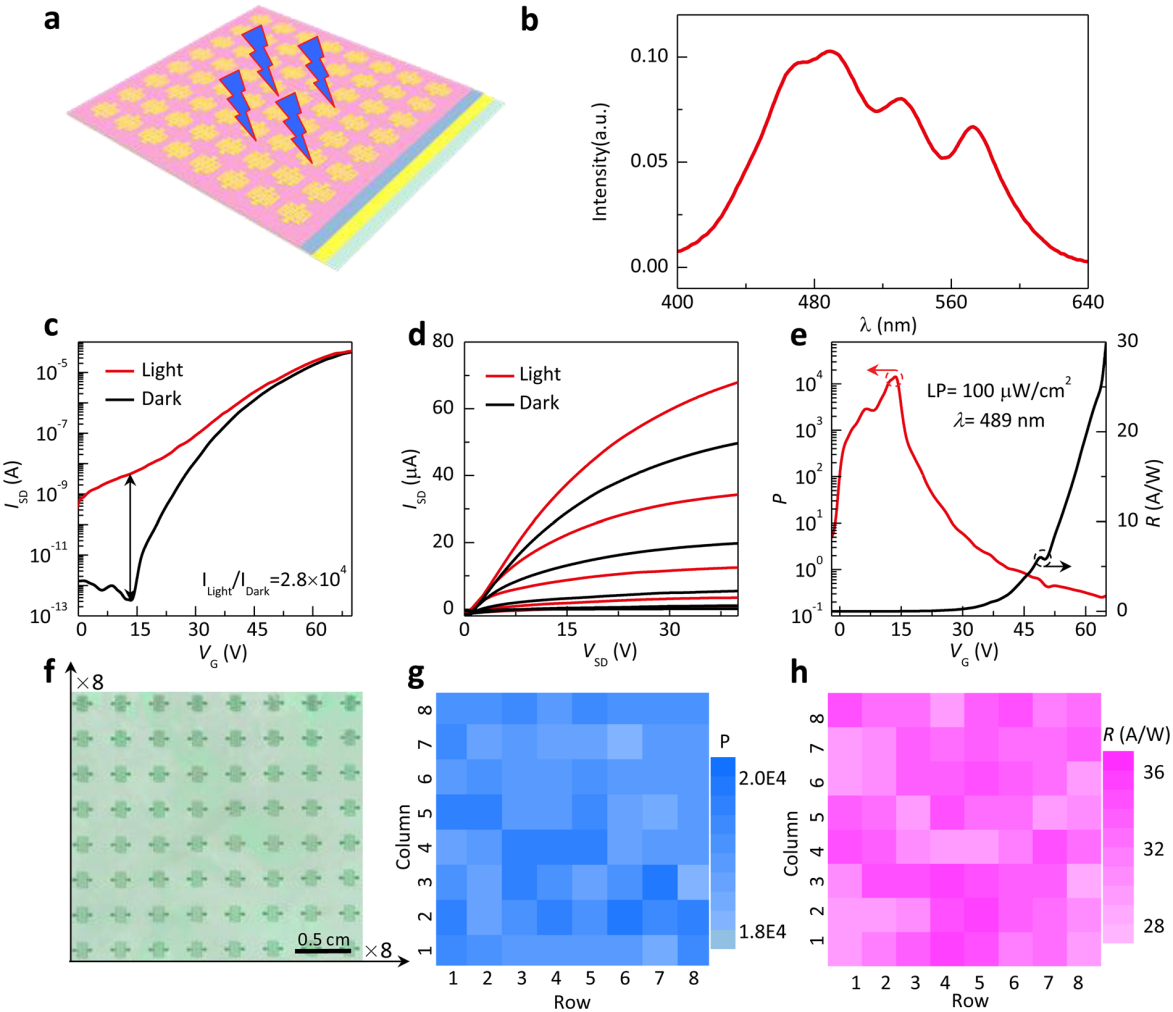
Optoelectronic properties of the PTCDI-C_13_H_27_ thin-film phototransistor. (**a**) Schematic image of the transistor under illumination. (**b**) Absorption spectra of the PTCDI-C_13_H_27_. (**c**,**d**) Typical transfer and output characteristics of the PTCDI-C_13_H_27_ thin-film phototransistor measured in the dark and under monochromatic light irradiation (wavelength: 489 nm, light power density: 100 μW cm^−2^). (**e**) *R* and *P* as a function of *V*_G_. (**f**) Photograph of the 8 × 8 PTCDI-C_13_H_27_ thin-film phototransistor array. (**g**,**h**) *R* and *P* mapping of the phototransistor array.

Figure 3 illustrates the typical photoresponse behaviors of the PTCDI-C_13_H_27_ thin film transistors in the flat state. The photoresponse characteristics of the device were measured by directly shining the monochromatic light from the top of the device as schematically shown in Fig. 3a. The PTCDI-C_13_H_27_ presents a wide range of absorption from 400 to 640 nm, mainly in the visible region (Fig. 3b). From the long wavelength edge, the bandgap of PTCDI-C_13_H_27_ is estimated to be only around 1.93–2.02 eV, which agrees well with the reference value (2.0 eV)^32^. Due to the narrow energy bandgap, these devices can be easily excited by light and a number of photogenerated charge carriers can be created under illumination^33^. Fig. 3c,d respectively show the transfer and output characteristics of the PTCDI-C_13_H_27_ OPT in dark and under illumination with the wavelength of 489 nm (light power density: 100 μW cm^−2^). The device exhibits a dramatically increase in the *I*_SD_ under illumination due to the absorbed photogenerated carriers to boost the current. From Fig. 3c, we calculated two important parameters of OPTs, namely, photosensitivity (*P*) and photoresponsivity (*R*), based on the following fundamental equations:

1 $$P=\frac{{I}_{light}-{I}_{dark}}{{I}_{dark}}$$

2 $$R=\frac{{I}_{ph}}{A{P}_{inc}}=\frac{{I}_{light}-{I}_{dark}}{A{P}_{inc}}$$

where *I*_ph_ is photo-current, *I*_light_ is drain current under illumination, *I*_dark_ is drain current under dark, *A* is effective device area, and *P*_inc_ is incident illumination power density^34^. *P* and *R* values as function of gate voltage (*V*_G_) are plotted in Fig. 3e. When the OPTs are in the off state under low gate voltage, the highest *I*_light_/*I*_dark_ exceeds 10^4^. When transistors are turned on by applying higher *V*_G_, the *I*_light_/*I*_dark_ decreases because of the increased dark current. And the maximum *R* value was calculated at 30.73 A W^−1^. These indicate that phototransistors can utilize this gate effect to modulate *P* and *R* values according to the requirement of practical applications^4^. In order to show the uniformity of properties of our OPTs, an OPT array of 64 transistors was fabricated (Fig. 3f). As shown in Fig. 3g,h, the color maps of the *P* and *R* of the OPT array present good uniformity with 100% yield. In all OPTs, *P* is higher than 10^4^ and *R* is higher than 28 A W^−1^. This *P* value is sufficiently high to be applied in in-cell touch screens and photodetectors^35^. These results indicate potential applications of PTCDI-C_13_H_27_ in low-cost flexible organic optoelectronics.

**Figure 4:**
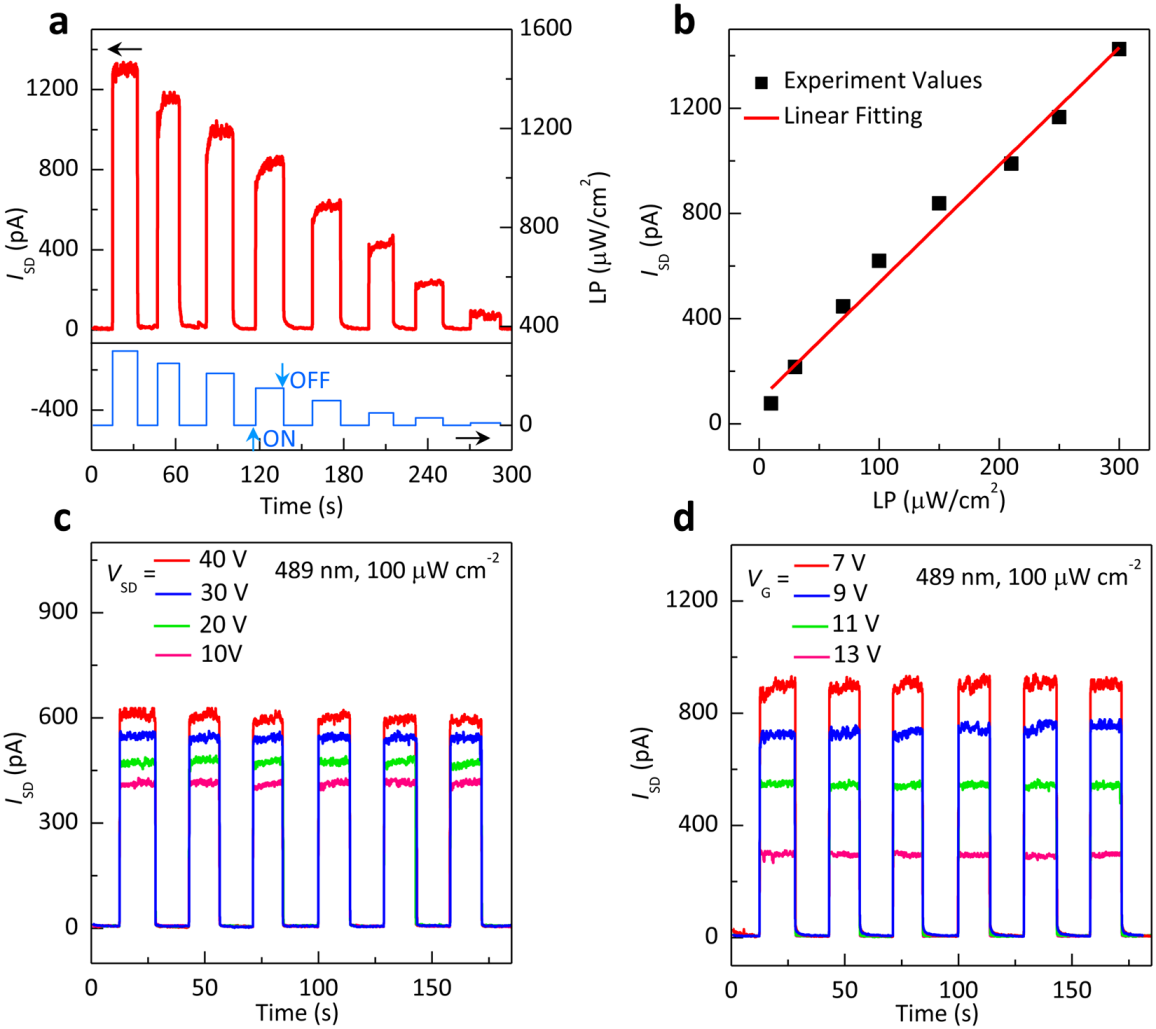
Dynamic photoresponse behavior of the PTCDI-C_13_H_27_ thin-film phototransistor. (**a**) Dynamic photoresponse under different irradiation (10, 30, 50, 100, 150, 210, 250, 300 μW cm^−2^) at fixed *V*_G_ = 9 V. (**b**) Light power density dependence of the photocurrent. (**c**) Dynamic photoresponse behavior of PTCDI-C_13_H_27_ OPT with different *V*_G_ at fixed *V*_SD_ = 30 V under the irradiation of 100 μW cm^−2^. (**d**) Dynamic photocurrent response of PTCDI-C_13_H_27_ OPT with different *V*_SD_ at *V*_G_ = 9 V.

Optical switch and signal amplifier are the critical applications for phototransistors^36,37^. Therefore, the dynamic photoswitching properties of the PTCDI-C_13_H_27_ thin film phototransistor under monochromatic light irradiation were further investigated. Figure 4 shows the dynamic photoresponse behaviors of the PTCDI-C_13_H_27_ thin film phototransistors. When the incidence light is turned on, the current of the PTCDI-C_13_H_27_ thin film device shows the obvious increase during the switch-on state of the optical power. On the contrary, the current rapidly recovers to the original value when the light is turned off. Figure 4a gives the real-time current response to the dynamic switches with the continuously decreased light intensity from 300 to 10 μW cm^−2^ with a fixed wavelength at 489 nm. A pronounced change in the current was observed under on/off switching of the light. All of the phototransistors show the clear photoresponse without any retardation effects, indicating that our PTCDI-C_13_H_27_ thin film phototransistor can serve as an optical switch. Further, Fig. 4b shows that the photocurrent linearly increases with the increasing incident light intensity. The remarkable increased photocurrent suggests the high photoresponse capability of our phototransistors. The photon energy is absorbed by PTCDI-C_13_H_27_ thin film under light excitation and a large number of photogenerated charges are generated that contribute to carrier transport^33^. The light intensity can act as an independent variation to modulate the current. Further, the dynamic photoresponse curves of the OPTs are plotted as a function of time at different *V*_G_ (Fig. 4c) and *V*_SD_ (Fig. 4d) at a fixed irradiation (λ = 489 nm, light power density = 100 μW cm^−2^). The photocurrent increases with the increasing *V*_G_ at fixed *V*_SD_ (*V*_SD_ = 30 V), and increases with the increasing *V*_SD_ at fixed *V*_G_ (*V*_G_ = 9 V), which show the modulation of gate voltage and source-drain voltage on electronic signal. The gate voltage is considered to provide an efficient route for the charge dissociation in the OPT devices^38^. Higher drain voltage effectively draws electrons to weaken the recombination of photogenerated carriers^14,38^. The reproducible photoswitch function in signal amplification and photodetection provides the OPTs potential applications in cost-effective flexible organic optoelectronics.

**Figure 5:**
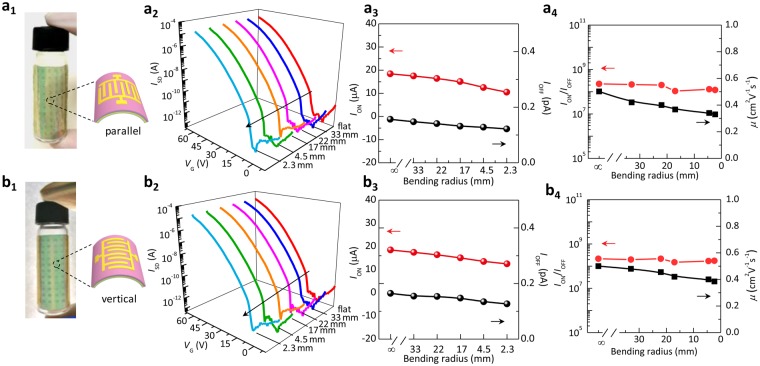
Typical photograph and schematic images, typical transfer curves, *I*_ON_, *I*_OFF_, mobility and *I*_ON_/*I*_OFF_ of the device at strain bending (**a**_**1**_**–a**_**4**_) parallel and (**b**_**1**_–**b**_**4**_) vertical to channel length. The bending radius is from 3.3 to 0.23 cm.

Organic electronics and optoelectronics are considered as key components of wearable smart electronics due to their flexibility and light weight. Therefore, flexible and conformable OPTs have attracted increasing interest in this field^1–4^. As shown in Fig. 1, our OPTs show good flexibility and extremely low thickness so that they can well conform onto various surfaces. Here, to show the application potential of the OPTs in wearable and portable optoelectronics, the OPT array is adhered onto the curved surfaces with different bending curvature by mechanical transferring. Figure 5 shows the typical photoresponse behaviors of the OPTs on different curved objects. The surface strain can be given by: *ε* = *t/*2*r*^39^, where *t* is the thickness of the flexible OPT, and *r* is the radius of bending curvature. According to this equation, the thickness (*t*) of the device determines the degree of flexibility. Lower thickness, smaller the stress. Therefore, the extremely thin device thickness presents an outstanding advantage for conformable devices. To explore the effect of bending degree on the device performance, the different bending radius from 3.3 to 0.23 cm (3.3, 2.2, 1.7, 0.45, 0.23 cm) was employed.

When the device is bent in different directions, the bending deformation states in the conductive channel are different. It is possible that such a bending state difference will affect the device performance, since the carrier transport is limited in a few molecular layer in the channel^40,41^. In order to explore the possible effect of the adherence direction on the device performance, the OPTs were transferred onto cylindrical objects in different adherence directions, i.e., with bending surface respectively parallel and vertical to channel length, as shown in Fig. 5a_1_,b_1_. The corresponding typical transfer curves in dark with the changed bending radius are respectively shown in Fig. 5a_2_,b_2_. Figure 5a_3_,b_3_,a_4_,b_4_ respectively give the dependence of the on-state current, the off-state current, the ON/OFF current ratio, and the mobility on the bending radius. The bending radius ranges from 3.3 to 0.23 cm. Figure 5 shows the weakly decreased on-state current, off-state current, and mobility, and almost unchanged ON/OFF current ratio. Three research groups have shown the bending results of the flexible device in the two different directions^42–44^. All of them show the decreased mobility with the bending in two different directions. The mobility change in the two different bending directions in our work is in good agreement with their reports. These results confirm that the freely bending of the device in different directions at fixed bending radius does not cause the mobility difference.

**Figure 6:**
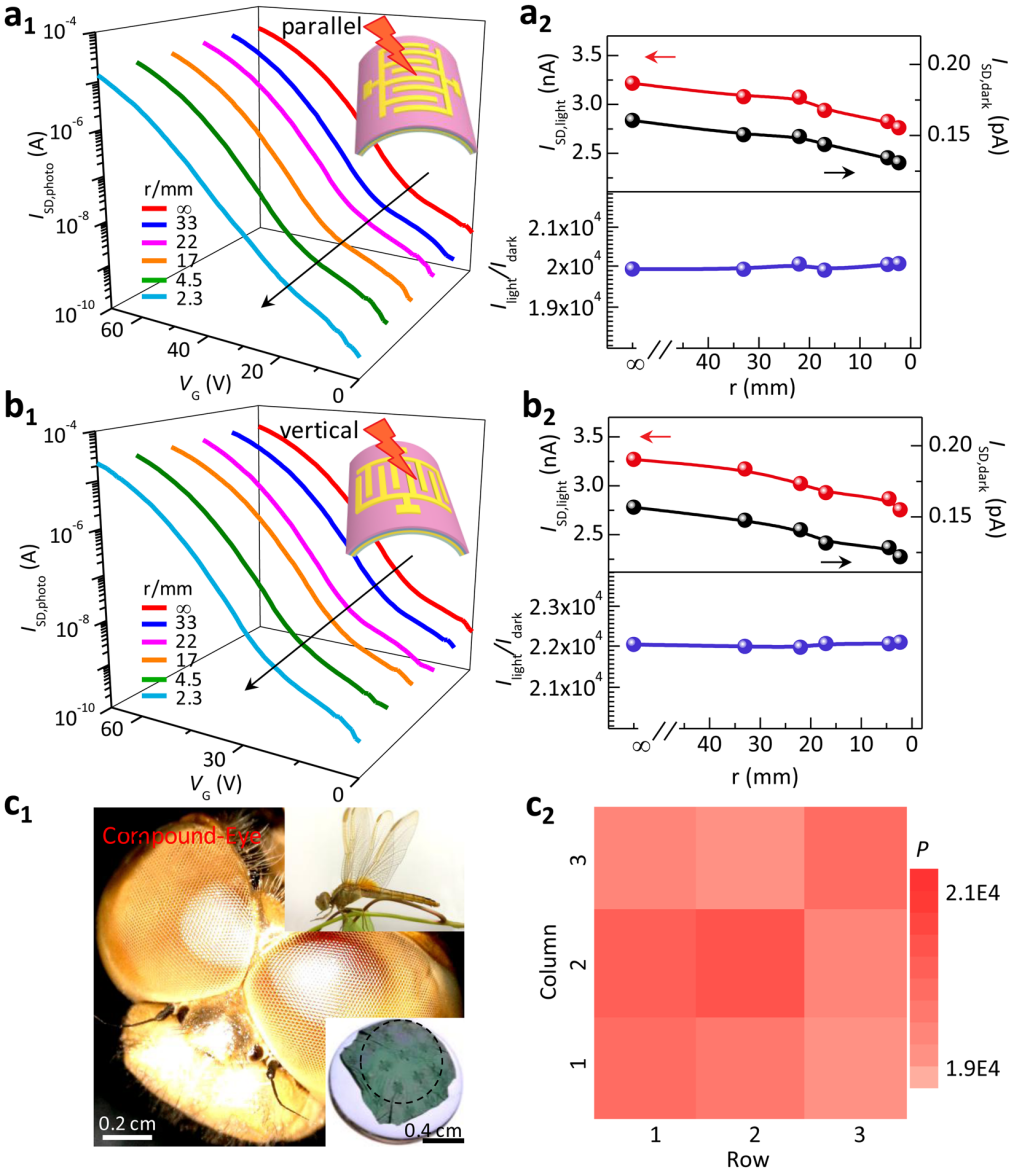
Photoresponse performance of the conformal PTCDI-C_13_H_27_ phototransistor array on different curved objects. (**a**_**1**_,**a**_**2**_,**b**_**1**_,**b**_**2**_) Typical photograph and schematic images (inset), typical transfer characteristics under illumination, *I*_light_, *I*_dark_ and *I*_light_*/I*_dark_ of the device at strain bending parallel and vertical to channel length, respectively. The bending radius is from 3.3 to 0.23 cm. (**c**_**1**_) Optical microscopy image of the compound eyes from a real dragonfly eye. The right-bottom optical microscopy image in the inset shows a real device array adhered on a hemisphere lens with the bending radius of 0.64 cm. (**c**_**2**_) Photosensitivity mapping with a 3 × 3 OPT array as shown in the inset of Figure c1. The wavelength of illumination is 489 nm and light power density is 100 μW cm^−2^.

The corresponding typical transfer curves under illumination with the changed bending radius are respectively shown in Fig. 6a_1_,b_1_. Figure 6a_2_,b_2_ respectively give the dependence of photocurrent, dark current, and photosensitivity on the bending radius. Eventhough the photocurrent and dark current of the PTCDI-C_13_H_27_ thin film phototransistor show the slight decrease with the decreased bending radius, the device still remains the stable photosensitivity as high as 10^4^. From our experiments, it can be concluded that the bending of the device weakly affects the mobility but almost does not affect the photosensitivity. The photosensitivity of the device on curved surfaces remains almost unchanged as their flat state, showing the high operational stability of our ultrathin OPTs under bending conditions (Fig. 6a_2_,b_2_). Further, it is found that the thickness of the semiconductor layer from 30 to 60 nm did not show the obvious mobility change for our devices (Fig. S2). This result is similar to the previous reports^45^. Our device thickness is ~830 nm. Here, the bending strain is mainly determined by the insulator thickness (~380 nm) and the supporting layer thickness (~350 nm) according to *ε* = *t/2r*^6^. Therefore, the thickness change of the semiconductor layer (30 and 60 nm) does not affect the device performance dramatically (Fig. S3).

Figure 6c_1_,c_2_ shows the potential applications of our conformal PTCDI-C_13_H_27_ thin film phototransistor array as the photodetection system of curved artificial compound eye^46^. Curvilinear photodetector array enables a wide-angle field of view, which is one of essential components of the curved artificial compound eyes to realize panoramic perception^46,47^. Figure 6c_1_ presents the compound eyes of a real dragonfly. As the photoreceptive component of curved compound eye camera, it is essential that the photo detectors with a large-scale array remain high conformability onto the curved sphere^5^. The right-bottom inset of Figure 6c_1_ shows our PTCDI-C_13_H_27_ thin film phototransistor array well adhered onto a hemispherical lens (*r* = 0.64 cm) with close contact, and Figure 6c_2_ shows its color map of the photosensitivity *P*. The highest *P* value exceeds 10^4^, and the average value is 1.9624 × 10^4^ with a standard deviation of 0.0382 × 10^4^. Our obtained *P* value is very appealing among *n*-type OPTs. Table 1 summarizes the photosensitivity of the reported *n*-type OPTs. For comparison, the photosensitivity values of our flat and conformal devices on 2D cylinder and 3D hemisphere, are also shown in Table 1. Compared with the previously reported results, our ultrathin flat device shows the photosensitivity as high as 2.8 × 10^4^ under illumination of only 100 μW cm^−2^. This value is higher than most of reported results, and is in a class with the highest photosensitivity for perylene diimide derivatives as shown in Table 1^23,34,36,37,40–44^. Furthermore, the previously reported *n*-type OPTs were fabricated on the rigid SiO_2_/Si, glass, or flexible PET, and all measurements were carried on the flat state. In comparison, our device not only shows the performance on bending surfaces, but also shows the photosensitivity over 2 × 10^4^. Such a high performance at bending state also presents promising application potential of our conformable air-stable *n*-type PTCDI-C_13_H_27_ OPTs in wearable and portable optoelectronics. It is worth mentioning that this photoresponse parameter keeps nearly unchanged as it flat state. That is to say, our phototransistor arrays have high photosensitivity, good uniformity, and high stability on 3D curved surface, indicating a wide variety of novel applications such as bionic eye that is not possibly realized on planar rigid supporting.

**Table 1:** Summary of performance of *n*-type organic phototransistor.

Semiconductor Material	State(Substrate)	P	Light Source (nm)	Intensity (μW cm^−2^)	Ref.
PDI-Cn	flat(SiO_2_/Si)	^(a)^10^3^	580	7.06	^34^
		^(b)^9			
		^(c)^100			
BPE-PTCDI	flat(SiO_2_/Si)	4.96 × 10^3^	green	113000	^23^
PTCDI-C_13_H_27_	flat (PET)	4 × 10^4^	532	22200	^23^
PDIF-CN2	flat(SiO_2_/Si)	5 × 10^3^	white light	5060	^40^
BPE-PTCDI/rGO	flat(SiO_2_/Si)	>10	640	17600	^41^
PTCDI-C8	flat(SiO_2_/Si)	>100	—	15000	^36^
EH PDI	flat(SiO_2_/Si)	63.82	525	91060	^42^
F16CuPc(thin-film)	flat(BPDA-ODA)	300	white light	5660	^43^
F16CuPc	flat(glass)	79	white light	5980	^37^
NDI(2OD)(4tBuPh)-DTYM2 PTCDI-C13	flat(SiO_2_/Si) flat(PVA)	1.1 × 10^7^ 2.8 × 10^4^	white light 489	107 100	^44^ Our work
	2D conformal (PVA) 3D conformal (PVA)	^(d)^2.2 × 10^4^ 2 × 10^4^			

^(a)^Thin film, ^(b)^multifib, ^(c)^monofib, ^(d)^average value at different bending radius^48–51^.

In summary, we fabricated the conformal *n*-type OPT array based on air-stable PTCDI-C_13_H_27_ and PMMA dielectric. The ultrathin thickness of devices only at ~830 nm makes the transistor array realize good adherence onto different curved objects. Large-area OPT array shows excellent electrical properties in dark state with the mobility as high as 0.58 cm^2^ V^−1^ s^−1^, the extremely high on/off ratio over 10^9^, and high stability in air atmosphere. When the OPT is transferred onto cylindrical objects with the surfaces bent respectively parallel and vertical to channel length, it is found that the performance changes, including on-state current, off-state current, ON/OFF ratio, and mobility, are similar although the conductive channel region presents the different bending deformation. With the decreased bending radius on different curved surfaces, both the dark current and light current of the conformal device present the weak decrease. However, the synchronous decrease of the dark and light current makes the photosensitivity remain unchanged and still remain at >10^4^ when the device is adhered on the objects with different curved radius. These results not only show the good photosensitivity consistency when our device array is freely adhered on the curved objects, but also the good uniformity of the optoelectronic performance and the high photosensitivity upon illumination as weak as 100 μW cm^−2^ present the promising application potential of our conformable air-stable *n*-type PTCDI-C_13_H_27_ OPT array, for example, as the photodetection system of the curved artificial compound eyes for wide-angle field of view.

## Methods

### OTFT Fabrication

Materials preparation and device fabrication: Heavily doped silicon wafers were used as substrate for ultra-flexible OPTs. These substrates were cleaned, subsequently were modified by a self-assembled octadecyltrichlorosilane (OTS) layer (Acros, 95%). The typical bottom-gate top-contact configuration was employed for the fabrication of OPTs. First, the aqueous solution of PVA was spin-coated on a Si substrate, followed by annealing under vacuum. Second, 30-nm-thick gold (Au) was thermally deposited as bottom-gate electrode. Third, the polymethyl methacrylate (PMMA) solution in anisole was spun on the top of Au gate electrode as dielectric layer at 3500 rpm and then the resulting sample was annealed in a stove to remove the residual solvent. Fourth, 30-nm-thick PTCDI-C_13_H_27_ thin film was deposited through vacuum thermal evaporation at a pressure of 2 × 10^−4^ Pa at the substrate temperature of 45 °C. Finally, 30-nm Au was deposited with the shadow mask and served as source and drain electrodes. The flexible conformal OPT device array was obtained by mechanically peeling the whole device array from the Si substrate with a hollowed-out 3 M tape (Scotch) in the help of a tweezer. All experiments were conducted on the lab instrument.

### OTFT Measurements

The electrical and photoelectrical properties of the phototransistor were measured by using a semiconductor parameter analyzer (Keithley 4200 SCS). The field-effect mobility (*μ*) and current on/off ratio (*I*_on_/*I*_off_) were extracted from the transfer characteristic curves. In the saturation regime, the mobility was calculated by the following equation: *I*_SD_ = *C*_*i*_*µ*(*W*/2 *L*)(*V*_G_ − *V*_T_)^2^. For the photoelectrical properties of PTCDI-C_13_H_27_ devices, a xenon lamp (HSX-UV300, NBeT) was employed as a light source, and the illumination intensity was measured by an optical power meter (No.1918-R, Newport). The light was illuminated from the top side of the device. And all devices were characterized in ambient atmosphere at room temperature.

## Electronic supplementary material

Supporting information
