# Supplementary material for: Ultrathin Air-Stable n-Type Organic Phototransistor Array for Conformal Optoelectronics
Source: Sci Rep. 2018 Nov 9;8:16612. doi: 10.1038/s41598-018-35062-7 (PMC6226476; doi:10.1038/s41598-018-35062-7)
Supplement: Supplementary file 1 — Supporting information [file 41598_2018_35062_MOESM1_ESM.doc]

Ultrathin Air-Stable n-Type Organic Phototransistor Array for Conformal Optoelectronics

Meiling Liu^1^, Haiting Wang^1^, Qingxin Tang*, Xiaoli Zhao, Yanhong Tong, and Yichun Liu*

Key Laboratory of UV Light Emitting Materials and Technology under Ministry of Education, Northeast Normal University, Changchun 130024, P. R. China

*Corresponding authors: Prof. Q. Tang and Prof. Y. Liu

^1^These authors contributed equally to this work.

E-mail address: tangqx@nenu.edu.cn; ycliu@nenu.edu.cn

Tel./fax: +86-431-85099873.

Figure S1: Typical multimeasured transfer characteristic curves for the PTCDI-C13 OTFTs devices with (a_1_, a_2_)PMMA, (b_1_, b_2_) PVA dielectrics. The well overlapped curves show the advantage of PMMA as dielectric.

Figure S2: Figure R4: Device mobility as function of thickness of semiconductor layer. The almost unchanged mobility with the semiconductor thickness can be observed.

Figure S3.Transfer curves at flat and bent state with different semiconductor layer thickness respectively at 30 and 60 nm. The calculated normalized mobility of the OTFT (the ratio of the mobility at bent state to the flat state) at the two different thickness is almost unchanged, suggesting the bent-induce relative change of mobility is independent to the thickness.
